# Rice plants overexpressing *OsEPF1* show reduced stomatal density and increased root cortical aerenchyma formation

**DOI:** 10.1038/s41598-019-41922-7

**Published:** 2019-04-03

**Authors:** U. Mohammed, R. S. Caine, J. A. Atkinson, E. L. Harrison, D. Wells, C. C. Chater, J. E. Gray, R. Swarup, E. H. Murchie

**Affiliations:** 1 Division of Plant and Crop Science, School of Biosciences, University of Nottingham, Sutton Bonington campus, LE12 5RD Nottingham, UK; 2 Department of Molecular Biology and Biotechnology, University of Sheffield, Western Bank, S10 2TN Sheffield, UK

**Keywords:** Plant physiology, Flooding, Stomata

## Abstract

Stomata are adjustable pores in the aerial epidermis of plants. The role of stomata is usually described in terms of the trade-off between CO_2_ uptake and water loss. Little consideration has been given to their interaction with below-ground development or diffusion of other gases. We overexpressed the rice *EPIDERMAL PATTERNING FACTOR1* (*OsEPF1*) *to* produce rice plants with reduced stomatal densities, resulting in lowered leaf stomatal conductance and enhanced water use efficiency. Surprisingly, we found that root cortical aerenchyma (RCA) is formed constitutively in *OsEPF1*OE lines regardless of tissue age and position. Aerenchyma is tissue containing air-spaces that can develop in the plant root during stressful conditions, e.g. oxygen deficiency when it functions to increase O_2_ diffusion from shoot to root. The relationship with stomata is unknown. We conclude that RCA development and stomatal development are linked by two possible mechanisms: first that reduced stomatal conductance inhibits the diffusion of oxygen to the root, creating an oxygen deficit and stimulating the formation of RCA, second that an unknown *EPF* signalling pathway may be involved. Our observations have fundamental implications for the understanding of whole plant gas diffusion and root-to-shoot signalling events.

## Introduction

Stomata act as gatekeepers that control gaseous diffusion between plants and the aerial environment. They play a critical role in responding to water deficit, maintaining high rates of gas exchange, regulating plant water use efficiency (WUE) and permitting nutrient uptake from roots via the transpiration stream. Stomatal apertures are responsive to environmental cues such as light, temperature, humidity and atmospheric CO_2_ concentration, and to intrinsically derived signals such as abscisic acid^1–3^. Stomatal development is also influenced by changes in the environment, resulting in altered stomatal density and/or size. These developmental adjustments, are controlled by both local and/or long distance signals, leading potentially to altered leaf gas exchange properties^4–6^. This high level of physiological and developmental plasticity makes stomata an attractive target for studies into the adaptation of crops to future environments.

Aerial and subterranean plant parts are functionally coordinated. First, stomata drive the process of water uptake from the roots by generating a substantial water potential differential through transpiration. This is accompanied by morphological coordination, for example there is a correlation between root length density and leaf area^7^. Recent evidence has also shown an association between high stomatal density and a larger root area^8^ suggesting that a sophisticated level of co-ordination exists between above and below ground organs. There is little evidence that stomata provide direct signals for root development even though such a function could be an efficient means of providing appropriate whole plant responses to sub optimal environmental (aerial) conditions. Nevertheless various genes and transcription factors have been implicated in the co-regulation of root and shoot development through the common systemic signals such as plant hormones (e.g auxin and cytokinins) and sugars^9–11^. Examples include the auxin-responsive *MORE AXILLARY GROWTH (MAX*)^12^ and the carotenoid derived *BYPASS1*^13^ genes.

In addition to photosynthesis and transpiration, a less well-characterised role for stomata is to provide oxygen to the roots via diffusion along specialised spongy tissue containing air spaces known as aerenchyma. Over a century ago, Sachs^14^ named two types of aerenchyma according to their method of formation: schizogeny results in cortical cell separation and differential cell expansion while lysigeny results in cell death and subsequent lysis of some cortical cells to form air space. Lysigenous aerenchyma is common among the cereal crops such as rice (*Oryza sativa* L.)^15,16^, maize (*Zea mays*)^17,18^, barley (*Hordeum vulgare*)^19,20^ and wheat (*Triticum aestivum*)^21^. Air spaces are formed specifically in the root cortex, after the content of the cells that form the lysigenous aerenchyma becomes partially digested, leaving the surrounding cell wall. Some small cells remain intact and these provide structural shape and nutrient transport (apoplastic and symplastic)^22^.

Aerenchyma tissue is formed either as part of normal plant development, or in response to abiotic stresses associated with hypoxia^23^, high temperature, drought^24,25^ or nutrient deficiency^26^. It also occurs in plants experiencing flooding or submergence which leads to enhanced internal diffusion of atmospheric and photosynthetic O_2_ from aerial regions to the flooded roots, thereby maintaining aerobic root respiration^27,28^. Additional functions of aerenchyma may include the diminution of carbohydrate and O_2_ consuming cells (the respiratory ‘burden’) and the ventilation of gases such as CO_2_ and methane from the roots to shoots^29^.

Unlike many other cereal crops, rice is highly tolerant to waterlogging. It has the ability to grow in water laden (paddy) fields where excessive water often limits oxygen diffusion in the soil^30^. Therefore, rice provides a good model system for studying the mechanisms that permit an efficient supply of air from the above ground canopy to the below ground root zone. However, the coordinating signalling pathways that permit the morphological changes associated with growth under such conditions are not well studied. Here we investigate the relationship between stomatal properties and root development using rice lines that overexpress *EPIDERMAL PATTERNING FACTOR 1 (OsEPF1) or EPIDERMAL PATTERNING FACTOR-Like 9* (*OsEPFL9*).

In Arabidopsis (*Arabidopsis thaliana*), at least three genes: *EPF1, EPF2* and *EPFL9*/STOMAGEN encode small secretory peptides that regulate stomatal development and spacing^31,32^. These peptides compete for the binding of the transmembrane receptor leucine-rich repeat receptor like kinases (LRR-RLKs), ERECTA (ER), ERECTA-LIKE (ERL) 1 and/or ERL2; interactions which are governed by the presence of the LRR-receptor-like protein TOO MANY MOUTHS (TMM)^31,32^ that acts as a specificity switch for the regulation of stomatal development^33^. EPF1 and EPF2 negatively regulate stomatal development; EPF2 primarily by preventing asymetric entry divisions early in the stomatal lineage; EPF1 primarily by preventing stomatal clustering later in the lineage. *EPFL9/STOMAGEN* on the other hand, which is expressed in the mesophyll tissues^34^, acts as a positive regulator by blocking EPF1 and EPF2 from binding to ERECTA family receptors (ERfs)^33,35^. By manipulating the expression of *EPF1, EPF2* and/or *EPFL9* it is possible to drastically alter stomatal density. Indeed, overexpression of *EPFL9* results in increased stomatal density^31^, whereas overexpression of *EPF1* or *EPF2* results not only in reduced stomatal density but also lowers leaf stomatal conductance and increases leaf and plant water use efficiency^4,36–38^.

Here we show that overexpression of *OsEPF1* in rice results in reduced stomatal density whereas overexpression of *OsEPFL9* increases stomatal density. Remarkably *OsEPF1* overexpression also leads to constitutively generated lysigenous aerenchyma regardless of soil media aeration or tissue oxygen content. Our experiments show that aerenchyma formation in the non-transgenic rice is normally dependent on the oxygen content of the root growth medium. These results demonstrate novel physiological and developmental links between root and shoot responses to abiotic stress.

## Methods

### Generation of the ectopically overexpressing OsEPF1 and OsEPFL9 rice lines

*OsEFP1* (Os04g54490) and *OsEPFL9* (Os01g68598) cDNA sequences were cloned into pCR-8/GW/TOPO entry vectors and then into pBRACT214 vectors via LR reactions^39^. pBRACT214 contains the maize (*Zea mays*) ubiquitin promoter and a hygromycin resistance cassette. Constructs were transformed into *Agrobacterium* by electroporation and introduced into rice through *Agrobacterium* mediated rice transformation of mature embryos (*Oryza sativa* L. japonica cvs. Nipponbare background) as described by Toki *et al*.^40^ with some modifications. The presence of the transgene was confirmed by PCR using transgene specific primer combinations (a ubiquitin promoter specific forward primer and EPF gene specific reverse primer) with expression levels verified by quantitative reverse transcriptase PCR (qRT-PCR, see Supplementary Table 1).

### Plant growth

Seeds were surfaced sterilized with 0.5% (v/v) sodium hypochlorite, rinsed thoroughly and subsequently germinated on moist filter paper in 9 cm petri dishes at 30 °C and a photoperiod of 13 hours for one week in an incubator (A1000, Conviron, Winnipeg). Germinated seedlings were transferred to a controlled environment chamber described previously^41^. Illumination was provided by 400 W metal halide lamps with a photoperiod of 12 hours, temperature of 28 °C/26 °C (day/night) and relative humidity of 70% ± 5% throughout. Photosynthetic photon flux density (PPFD) was 400 µmol m^−2^ s^−1^ at plant height.

For the water restriction experiment, surface sterilized seeds were placed directly on to germinating plugs on a module tray for 7–10 days before being transferred to 3 L pots containing rice growth compost which was a mixture of John Innes No. 1 (John Innes, Norwich UK) and Levington M3 (JFC Monro, Devon, UK) in a ratio of 50:50 (w:w) and a slow releasing fertiliser mix (Osmocote, Scotts, LLC, Ohio). Water restriction was applied at two growth stages; 5-weeks and 8-weeks after germination and lasted for 8 and 7 days respectively. Soil water measurements were taken daily using a theta-probe from Delta T (Cambridge, UK). Control (well-watered) plants and treated plants were randomised within the growth chamber.

For experiments using hydroponically grown plants, the germinating seeds were transferred into media with a full complement of nutrients in light proof 20 L tubs as described previously^41^. For oxygen-deficient conditions, no gas was applied to the containers. For oxygenated conditions, air was continually bubbled into the media using aquarium pumps. The dissolved oxygen level was measured using a portable oxygen meter (YSI Pro20 Xylem Inc, USA). Transformed and control plants were randomised within each tub (11 plants per tub) and the position of each tub was randomised within the growth chamber.

### Stomatal density

Stomatal impressions were made on mature, fully expanded leaves using dental resin (Coltene President Plus Jet, Colten Whaledent, Switzerland) as described previously^42^. Stomatal size (µm^2^) was calculated using ImageJ (v1.49) as the guard cell length × guard cell pair width^6^ from images collected at x40 magnification. Typically, five to six plants per genotype and four measurements per leaf were examined in the central portion of the leaf, between midrib and margin.

### Leaf gas exchange measurements

All leaf gas exchange measurements were taken on the uppermost, fully expanded leaf using an infra-red gas analyser (IRGA), Licor 6400XT (Licor inc, Illinois, Nebraska). Three types of measurements were made: single measurements of photosynthesis (A) at light-saturation (*A*_max_), Photosynthesis versus intercellular CO_2_ concentration (*C*i, *A*/*C*i) analysis and light response curves as described previously^41,43^. Except where noted, measurements were made under light saturated conditions. The block temperature was maintained at 28 °C, the flow rate 500 ml min^−1^ and 60–65% humidity. The CO_2_ concentration used was 400 µmol mol^−1^ (except where noted) and PPFD 1500 µmol m^−2^ s^−1^. Measurements took between 2 and 3 minutes to achieve stability. The leaf instantaneous water use efficiency (IWUE) and intrinsic water use efficiency values (iWUE) were obtained from the ratio of photosynthetic CO_2_ assimilation (*A*) to stomatal conductance (*g*_*s*_), and *A* to leaf transpiration (*E*) respectively.

### Leaf dehydration assays

The IRGA auto-logged measurements every 30 seconds in 8 week-old plants (conditions described above). Typically, immediately after the first steady state measurement was obtained, the leaf was excised from the plant at about 5 mm away from the chamber gasket. Logging of gas exchange values of the leaf in the chamber continued as the excised leaf underwent water loss. The values obtained were normalised on the initial pre-excision value. A second assay used the same principle but measured leaf weight progressively after excision. A fully expanded leaf of known area (5 cm^2^ in 4–5 week-old and 7 cm^2^ in 8 week old) was excised and immediately weighed at 15 second intervals for 10 minutes. RWC was measured as described previously^43^.

### Root sectioning and imaging

Root samples (3.5–4 cm in length) from 15, 30 and 60 day old plants were obtained from three different positions of the seminal and adventitious roots (2 cm, 6 cm and 13 cm from the root tip). The root samples were collected in water and were placed in custom-designed polylactic acid molds and embedded with 4–5% agarose gel as described in Atkinson and Wells^44^. 150–180 µm thick sections of agarose embedded roots were cut using a Vibrating Microtome Ci 7000smz-2 (Campden Instruments Ltd, England) with blade frequency of 65 Hz. Sections were stained with Calcofluor-white (0.3 mg ml^−1^) for 1–3 minutes and rinsed with deionised water.

Root sections were imaged using an Eclipse Ti CLSM confocal laser scanning microscope (Nikon Instruments Inc, USA) using excitation and emission wavelengths of 440 nm and 540 nm respectively. Open source image software packages Fiji^45^ and ImageJ were used for image processing. The Root Scan v2.0 software (Penn State, College of Agricultural Science, USA) was used to generate root anatomical primary measurements based on pixel number. It was also used to calculate secondary measurements (e.g. percentage of the cortical area made up of aerenchyma air spaces) as described in Burton *et al*.^46^. Root parameters such as root total length, average root diameter, root surface area and root volume were obtained from scanned images of roots using a commercial software package WinRHIZO v6.1 (Regent instruments, Canada) as described in Himmelbauer^47^.

### RNA extractions, cDNA synthesis and Reverse transcription (RT) and qRT-PCR

RNA extractions were performed on 8-day old above ground seedlings or 30 day old adventitious root samples. Spectrum™ Plant Total RNA Kit (Sigma-Aldrich, Dorset, UK) for seedlings, or TRIzol™ Reagent (Invitrogen, USA) for root samples were used to extract RNA. Both sets of samples were treated using the Sigma On-Column DNase I Digestion Set. For root samples, 15 µl of eluted RNA was mixed with 500 µl of Spectrum™ Plant Total RNA Kit binding buffer and the manufacturers protocol was subsequently followed. All samples were standardised to 100 ng µl^−1^ and cDNA synthesis was performed with 18 bp oligo dTs using M-MLV Reverse Transcriptase (Invitrogen, USA).

RT-PCR was performed using OneTaq® Quick-Load® 2X Master Mix with Standard Buffer (NEB, USA) using approx. 25 ng of cDNA. For qRT-PCR, analysis was performed using the Qiagen Rotor-Gene SYBR® Green PCR Kit on a Corbett RG-6000 real-time cycler (Qiagen, Hilden, Germany) using approx. 10 ng of cDNA. All primers used were tested using seedling cDNA to verify performance with qRT-PCR dilution curves produced for each primer pair. For seedling analysis, the Profilin (Os06g05880) housekeeping gene was used. For roots, the Profilin and TFIIEβ (Transcription initiation factor IIE, Os10g25770) genes were used (Supplementary Table 1). Data was interpreted as in Luna *et al*.^48^ and Caine *et al*.^4^.

### Root elongation rate

Five -day old seedlings were transferred into a sealed 12 × 12 cm petri plate with one side removed for the plant shoots to grow outside the plate. The plates were filled with liquid hydroponic media (as above) and positioned vertically (at 90° to horizontal). The position of the root tip was marked on the plate. The plates were transferred into a controlled growth room. Images were taken every 6 hr using an automated image acquisition system^49^. Distance travelled by root per unit time was then used for the calculation of average root elongation rate (within the plate)^50^.

### Statistical analysis

Statistical analysis was performed using GraphPad Prism 7.01 for Windows (La Jolla, CA, USA) and GenStat for windows, 17^th^ Edition (VSN International Ltd.). Analysis of variance (ANOVA, one-way and two-way) with Tukey’s multiple comparison procedure were used except where indicated. Student’s t-tests were used in comparisons of RWC, SWC and leaf temperature. Pearson correlation analysis was applied.

## Results

### EPF/L overexpression results in altered stomatal density and size

**Figure 1:**
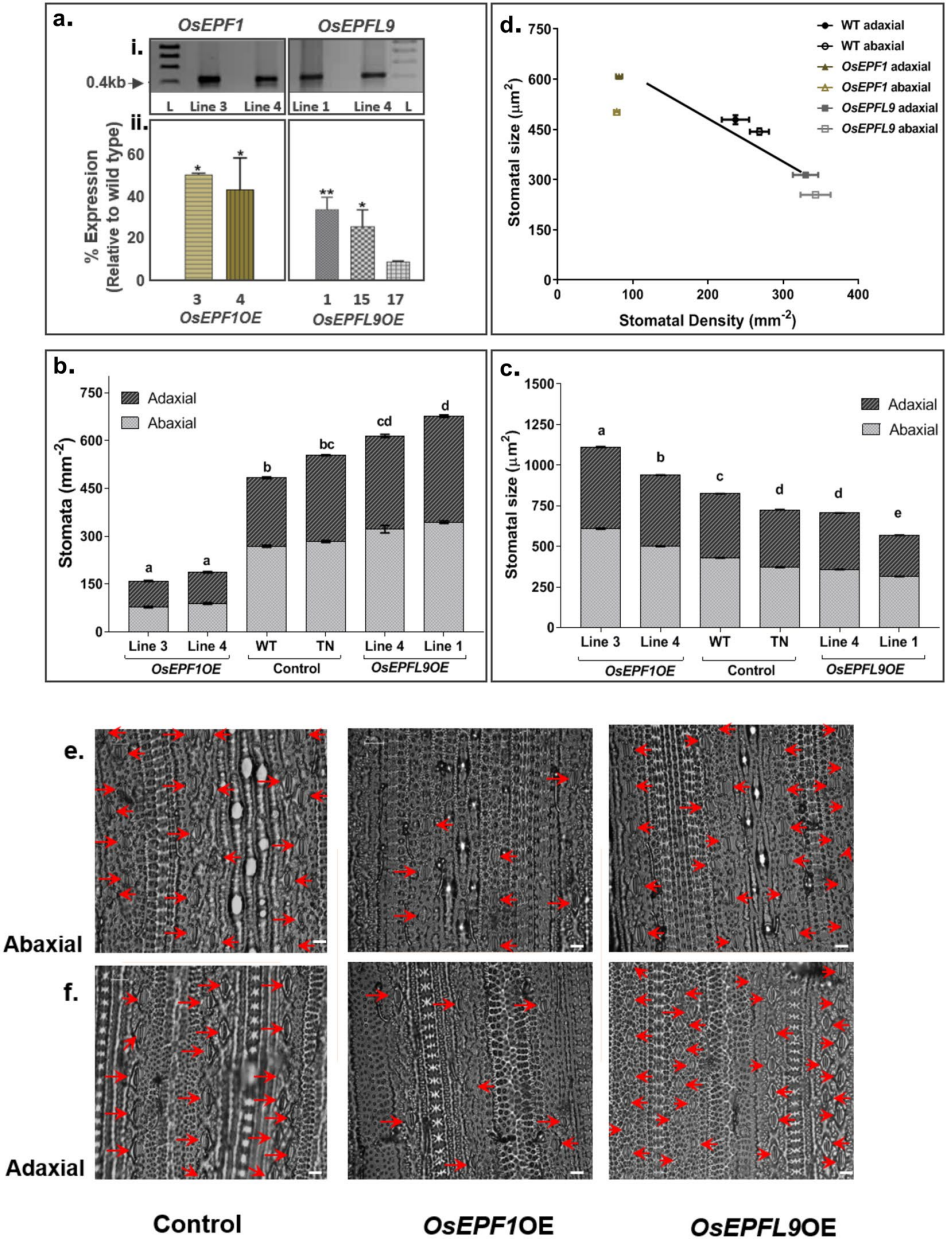
Overexpression of *OsEPF/L* genes in rice causes alterations in stomatal size and density. (**a**) Expression analysis showing overexpression of *OsEPF1* and *OsEPFL9* genes in independent transgenic lines, ai. Reverse transcription – PCR of *OsEPF1*OE (line 3 and line 4) and *OsEPFL9*OE (Line 1 and 4), L denotes ladder, aii. Quantitative RT-PCR of *OsEPF1*OE (line 3 and 4) and *OsEPFL9*OE (Line 1, 15 and 17). (**b**,**c**) Stomatal density (**b**) and stomatal size (**c**) measurements of 5 week old *OsEPF1* and *OsEPFL9* overexpression lines on the adaxial and abaxial leaf surfaces of a fully developed 5^th^ leaf. (**d**) The relationship between the mean of the stomatal sizes and mean of the stomatal densities of the adaxial (closed circles) and abaxial (open circles) surfaces. (**e**,**f**) Leaf impressions showing stomata distribution in untransformed wildtype (WT) controls and *OsEPF* overexpression lines on adaxial (**e**) and abaxial (**f**) surfaces. TN refers to (transformed) transgene negative plants. Data are represented as mean ± SEM. n = 4–6. Different letters indicate significant differences between the lines. (p < 0.05). Scale bar = 25 µm.

*OsEPF1* and *OsEPFL9* transgenic lines were produced through Agrobacterium mediated rice transformation^40^. The presence of the transgene in the primary transformants was checked by genomic DNA PCR which revealed that 21 out of 24 putative OsEPF1 overexpression lines and 17 out of 21 OsEPFL9 overexpression lines were transgene positive. Selected positive lines were initially checked for the expression of the transgene by RT-PCR and subsequently by qRT-PCR. Results showed that the expression level of *OsEPF1* (lines 3 and 4) and *OsEPFL9* (lines 1, 15 and 19) were significantly higher in these lines than the WT by about 50 and 35 fold, respectively (Fig. 1a).

Studies in Arabidopsis, barley and poplar (*Populus tomentosa*) show that the overexpression of *EPF/L* genes results in changes in stomatal density^31,51,52^. In our experiments with the Nipponbare cultivar of rice, *OsEPF1* and *OsEPFL9* overexpression (OE) lines were created and stomatal density was measured. These results are consistent with previous work indicating *OsEPF1* and *OsEPFL9* perform signalling roles in rice^4,53,54^. The two independent *EPF1* overexpression lines assessed both showed significant reductions in stomatal density (67% reduction in line 3 and 62% in line 4). For line 3 this equated to a 61% reduction on the adaxial leaf surface and a 71% reduction on the abaxial surface. For line 4 the reductions were slightly lower at 55% and 67% respectively. In contrast, overexpression of *OsEPFL9* resulted in a significant increase in stomatal density in both lines measured, with line 1 showing an overall increase of 28% (Abaxial, 22%; Adaxial, 35%) (Fig. 1b,e,f). Changes in density on both leaf surfaces were accompanied by increased stomatal size in *OsEPF1*OE lines and reduced stomatal size in the *OsEPFL9*OE lines (Fig. 1c). This strong inverse relationship between stomatal density and size (Fig. 1d), is consistent with results from a number of other species^36^. Interestingly, a recent study using *OsEPF1* overexpression in the IR64 rice cultivar displayed an opposite stomatal size effect in reduced stomatal density plants, with smaller rather than larger stomata^4^ forming, suggestive of different modes of regulation for stomatal size between Nipponbare and IR64.

### Altered stomatal density does not significantly alter photosynthesis in OsEPF1 or OsEPFL9 transgenic lines

**Figure 2:**
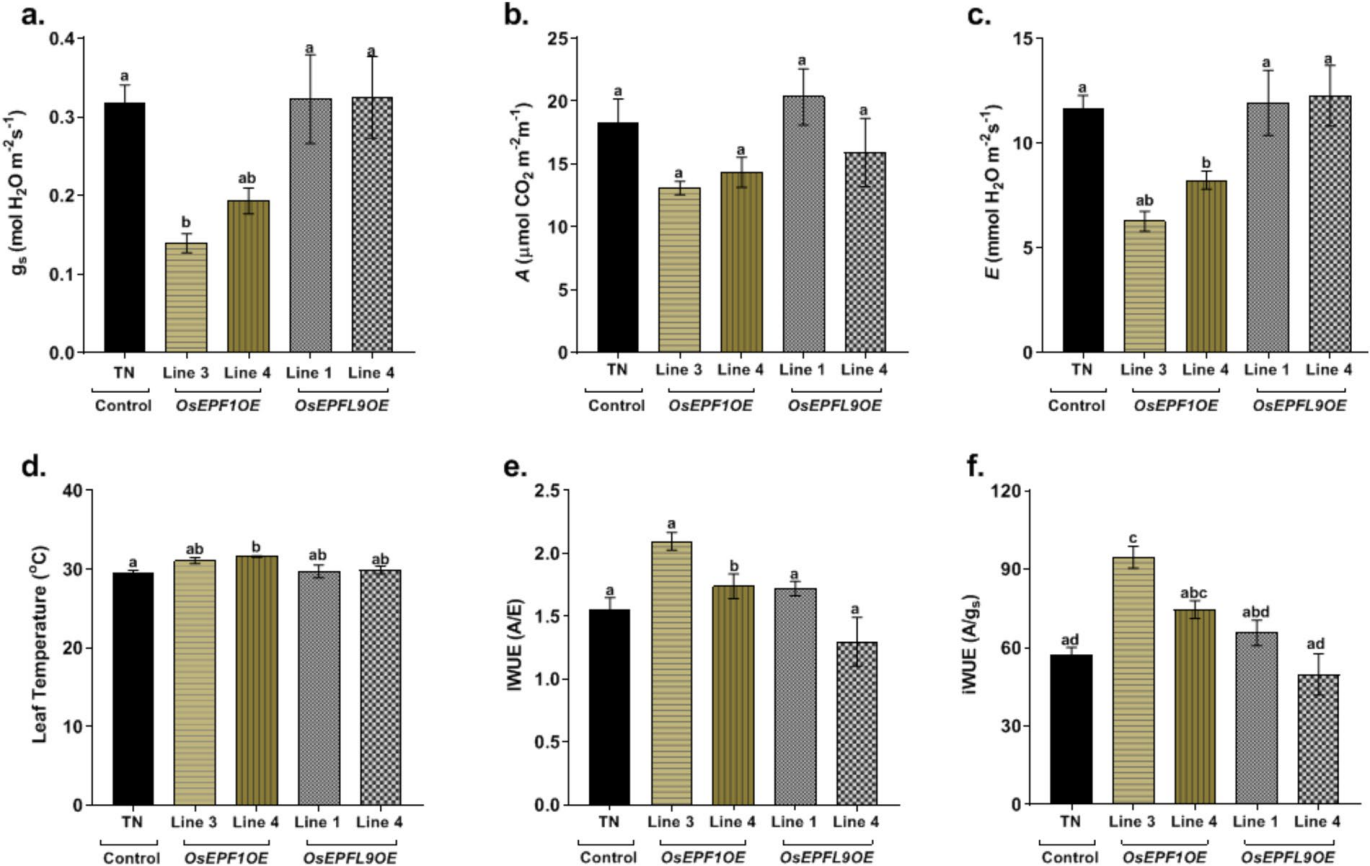
*OsEPF* overexpression lines show altered stomatal conductance and comparable photosynthesis to control plants. Five week old control plants and *OsEPF* overexpression lines were used for the analysis of (**a**) Stomatal conductance (*gs*), (**b**) CO_2_ assimilation rate (*A*), (**c**) Transpiration rate (*E*), (**d**) Leaf temperature, (**e**) Instantaneous water use efficiency (*A/E*), (**f**) Intrinsic water use efficiency (*A/gs*). All values are means ± SE (n = 4–5, Letters represent significant differences among the genotypes. P ≤ 0.05).

To understand how altering stomatal density alters leaf gas exchange properties we undertook IRGA measurements at 400 ppm CO_2_ on the leaves of 5 week-old plants (Fig. 2). Plants with lower stomatal densities (*OsEPF1*OE) had significantly reduced *g*_*s*_ and *E* comparative to transgene negative controls (P < 0.05). There was also a trend towards lower *A* in *OsEPF1*OE lines but this was not statistically significant. In comparison, *OsEPFL9*OE plants showed no significant differences in *A*, *g*_*s*_ or *E*. Calculation of intrinsic water use efficiency (iWUE, *A*/*g*_*s*_) revealed *OsEPF1*OE lines had significantly increased iWUE, but for *OsEPFL9*OE lines, values were comparable to controls. We conclude that water loss via *g*_*s*_ is reduced as a result of lowered stomatal density, and it is this change more so than adjustments in *A* that lead to improved iWUE. Light response curves confirmed that there were no differences in light compensation point or dark respiration between WT and either *OsEPF1*OE or *OsEPFL9*OE plants (Supplementary Fig. 1). Whilst WT and *OsEPF1*OE plants had similar quantum yield, *OsEPFL9*OE plants had significantly higher quantum yield than WT (P = 0.003) (Supplementary Fig. 1). Supplementary Fig. 2 shows that the differences in iWUE between *OsEPF1*OE and control were also present at low light intensities (<200 μmol m^−2^ s^−1^).

### Reducing stomatal density slows leaf dehydration, enhances drought tolerance and soil water conservation

**Figure 3:**
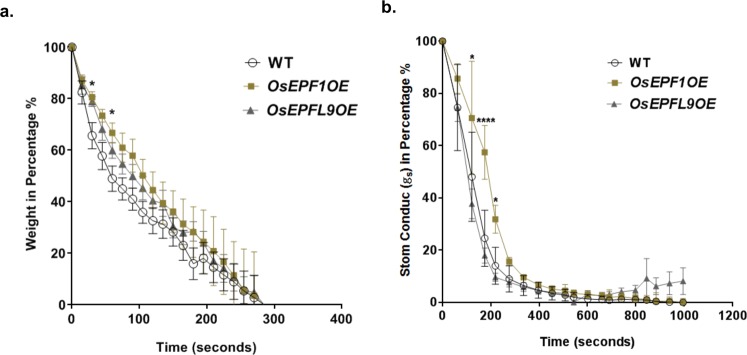
Reduced stomatal density slows leaf dehydration. Rapid leaf dehydration assay (leaf excision) of youngest fully expanded leaf on 8 week old plants. (**a**) The rate of water loss over time in an excised leaf (excised at 0 seconds). (**b**) Stomatal conductance in an excised leaf. The values were normalized to the initial value just prior to excision. Each data point represented as the mean and error bars indicate ± SEM. The asterisks represent the significant differences between the *OsEPF1*OE and the WT (*P ≤ 0.05 and ****P ≤ 0.0001). N = 4–6.

The effect of altered stomatal density on rapid water loss by leaf excision was investigated. Leaf weight declined most rapidly in the control plants, with *OsEPF1*OE plants showing a greater ability to retain water at 30 and 60 seconds after excision (P = 0.00458 and P = 0.0446 respectively) (Fig. 3a). The performance of *OsEPFL9*OE line did not significantly differ from WT. When *g*_*s*_ was assessed, *OsEPF1*OE leaves were slower to decline with significant differences at 120, 180 and 220 seconds after excission (P = 0.0109, P < 0.0001 and P = 0.0176 respectively), indicating that the rate of weight loss was due to a slower rate of water loss (Fig. 3b).

**Figure 4:**
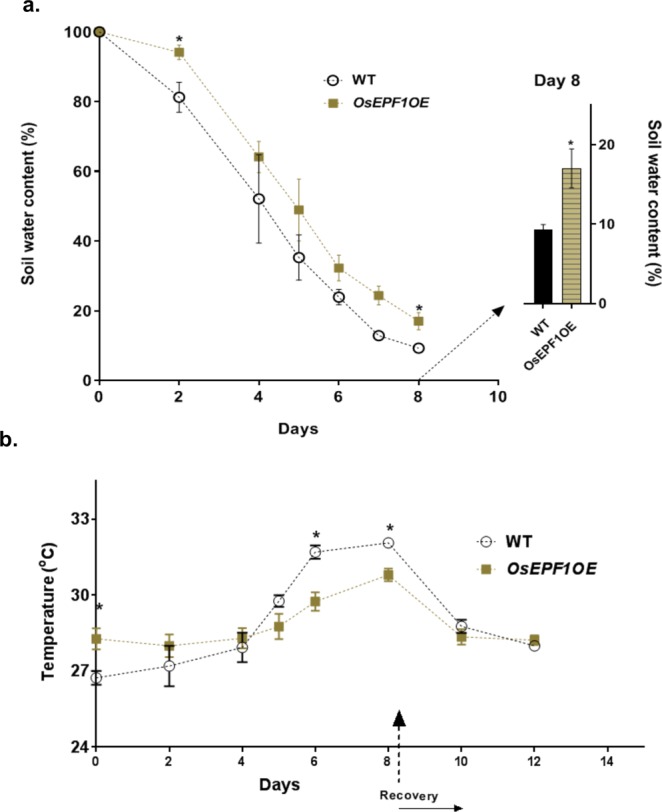
Reduced stomatal density improves soil water conservation. (**a**) Changes in soil water content in 5 week old plants over 8 days of drought stress. (**b**) Mean leaf temperatures during the drought stress and following re-watering compared to soil water at saturated state. Each data point represented as the mean and error bars indicate ± SEM. The asterisks represent the significant differences between the *OsEPF1*OE and the WT (*P ≤ 0.05). N = 4–5.

To further test if altered stomatal density affected responses to a water deficit, drought was imposed on the *OsEPF1*OE and WT plants at two growth stages (see methods). The soil water content (SWC) of plants droughted from 5 weeks post germination declined over the period of drought (Fig. 4a) and was consistently higher in *OsEPF1*OE than the WT throughout (P = 0.0315). On day 8 the SWC in *OsEPF1*OE was almost twice that of the control plants (P = 0.0474). Consistent with the finding that low stomatal density plants have lower *g*_*s*_ and *E*, leaf temperature was also monitored and found to be initially significantly higher in *OsEPF1*OE plants, but this was reversed by day 6 when the SWC dropped below 40% in all plants **(**Fig. 4b). Tiller number and whole plant leaf area were not significantly different between the WT and the *OsEPF1*OE lines (Supplementary Fig. 3). We conclude that the reduced *g*_*s*_ and *E* in *OsEPF1*OE lines led to greater soil water retention, which in-turn resulted in *OsEPF1*OE plants being able to maintain a higher rate of transpiration for longer, thereby preventing leaf temperatures from increasing as in WT under droughted conditions.

**Figure 5:**
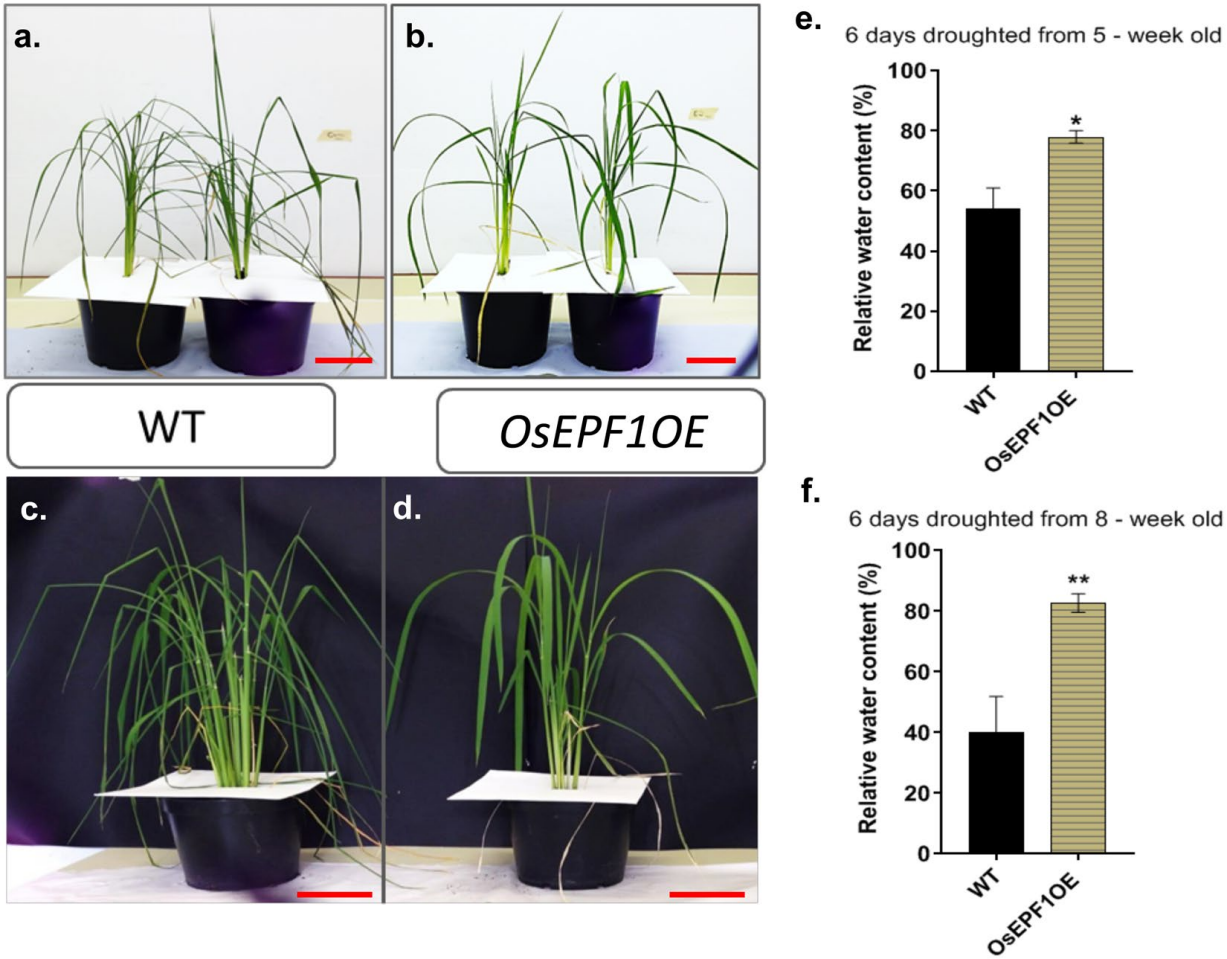
Reduced stomatal density improves leaf turgor and water conservation. *OsEPF1OE* plants show better leaf turgor and water retention than the WT leaves. (**a**–**d**) Representative 5 week (**a**,**b**) and 8 week (**c**,**d**) old (post germination) WT and *OsEPF1OE* plants on day 6 of drought stress. (**e**,**f**) Relative water content of 5 week (**e**) and 8 week (**f**) old (post germination) plants at day 6 of drought stress, normalised to the well-watered plants. The asterisk (*) represents the significant differences between the two treatments (*P ≤ 0.05 and **P ≤ 0.01) and error bars indicate SEM. Scale bar = 12 cm.

The drought treatment (Fig. 4) directly resulted in a reduction in leaf relative water content (RWC; Fig. 5) with less severe changes observed in *OsEPF1*OE plants. Under well-watered control plants (for both the 5 and 8 week old drought experiments) both WT and *OsEPF1*OE maintained approximately 95% leaf RWC. The RWC of all droughted plants was examined on day 6 of the drought with *OsEPF1*OE plants having significantly higher RWC than WT during both drought treatments (P = 0.0156 and P = 0.0058 respectively) (Fig. 5). Gas exchange was recorded over the 8 days of drought stress and recovery (Supplementary Fig. 4). Whilst typical drought-dependent declines in *A* and g_s_ were observed for both the WT and *OsEPF1*OE, significantly higher *A* and *g*_*s*_ values were observed in *OsEPF1*OE plants after day 5 (P = 0.0043 and P = 0.0372). This *g*_*s*_ response is consistent with our results indicating slower water loss in Fig. 3. Although not significantly different, *OsEPF1*OE plants also appeared to show more rapid rates of recovery than WT upon re-watering (Supplementary Fig. 4). Once again, these data indicate the importance of *g*_*s*_ and stomatal density in soil water conservation to support higher gas exchange values over periods of extended drought.

To further examine leaf gas exchange properties during the drought treatment, *A* vs *Ci* measurements were taken and curves fitted using a mechanistic model of *A*, and the biochemical components of *A*, *V*_cmax_ (maximum carboxylation rate of Rubisco) and *J*_max_ (maximum rate of RuBP regeneration) were estimated from a modified curve fitting model (Supplementary Fig. 5). There were no significant differences in *V*_cmax_ or *J*_max_, supporting the data in Fig. 2. Stomatal limitation on *A* (*L*_s_) was calculated^55^. The *L*_s_ can be viewed as the proportion of the value of *A* that results from stomatal resistance rather than other factors such as Rubisco activity or electron transport. The *L*_s_ was found to be significantly higher in droughted plants compared to well-watered, as expected during partial stomatal closure. During drought, the mean *L*_s_ values of *OsEPF1*OE plants showed a trend toward being lower than controls, but this was not significant (P = 0.4005). Differences in *L*_s_ were not necessarily expected, given that the stomatal conductance value during drought may be partially dependent on differences in soil water conservation. The equivalence of *L*_s_ between plant types under well-watered conditions is consistent with the observation above that lowered stomatal density reduces *g*_*s*_ to a greater extent than *A*.

### Overexpression of OsEPF1 enhances formation of root cortical aerenchyma independently of oxygen availability

**Figure 6:**
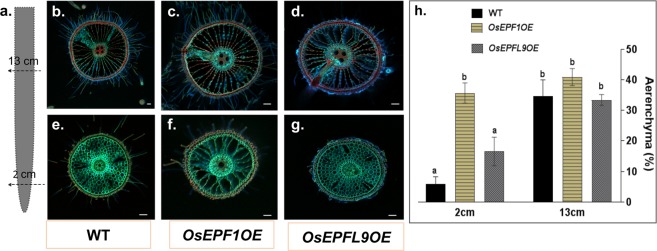
Overexpression of *OsEPF1* results in an increase in root cortical aerenchyma. Spatiotemporal formation of aerenchyma in 60-day old plants induced by constitutive overexpression of *OsEPF1* and *OsEPFL9*. (**a**) RCA was spatiotemporally evaluated in two positions; 2 cm and 13 cm from the roots tips. (**b**–**g**) Sections taken at 13 cm (**b**–**d**) and at 2 cm (**e**–**g**) of WT, *OsEPF1*OE and *OsEPFL9*OE roots. (**h**) Estimated aerenchyma formed in the two root regions. Different letters above the columns indicate significant differences between lines and sampling region (p < 0.05). All values are represented as the mean ± SEM. N = 4–7. Scale bar = 50 µm.

To test the role of *OsEPF1* and *OsEPFL9* overexpression on root cortical aerenchyma formation (RCA), the roots of the *OsEPF1* (line 3) and *OsEPFL9* (line 1) overexpression lines and the WT were evaluated. The seminal and adventitious roots of 15, 30 and 60 days old plants were examined for RCA formation. The effect of stomatal density and/or *OsEPF1* and *OsEPFL9* constitutive overexpression on the formation of RCA was evaluated in two positions; 2 cm and 13 cm from the roots tips (Fig. 6). Aerenchyma tissue could be observed in all the three lines tested (WT, *OsEPF1*OE line 3 and *OsEPFL9*OE line 1) at all three growth stages but was more widespread in *OsEPF1*OE.

Measurements of root length, diameter and biomass in 60 day old plants indicated no significant differences between root growth WT, *OsEPF1OE* and *OsEPFL9OE* plants (p < 0.05, Supplementary Fig. 6). We then tested the rate of root elongation using an automated image acquisition system^49^. The results show no significant difference between the WT and any of the transgenic lines in terms of root length and rate of root elongation up to 144 hours (the same sampling time point as the aerenchyma measurements). Furthermore, there were no differences in root length nor rate of seminal root elongation after 247 hours (Supplementary Fig. 7**)**. We therefore conclude that the root aerenchyma formation observed in the transgenic lines is caused by the over expression of *OsEPF1* rather than a developmentally induced change resulting from differences in the rate of root elongation.

We quantified formation of aerenchyma tissue by calculating the % of the cortical area consisting of air spaces using images of root cross-sections. At 2 cm from the root tip (Fig. 6H), 35.7% of the cortical cells in the *OsEPF1*OE lines were lysigenously digested, forming air spaces whereas *OsEPFL9*OE and the WT had only 16.5% and 5.8% air spaces respectively in this region. Thus, *OsEPF1*OE had 83.5% (P < 0.0001) more air space 2 cm above the root apex, in comparison with WT. At 13 cm from the root tip where the aerenchyma was fully established in all the three genotypes, there were no statistically significant differences observed. In *OsEPF1*OE roots, there was no difference in the percentage of aerenchyma found in the two regions that were sampled. Thus, we conclude that the RCA in *OsEPF1*OE lines was formed at the early stage of root organogenesis, whereas in the WT and *OsEPFL9*OE this occurred later.

**Figure 7:**
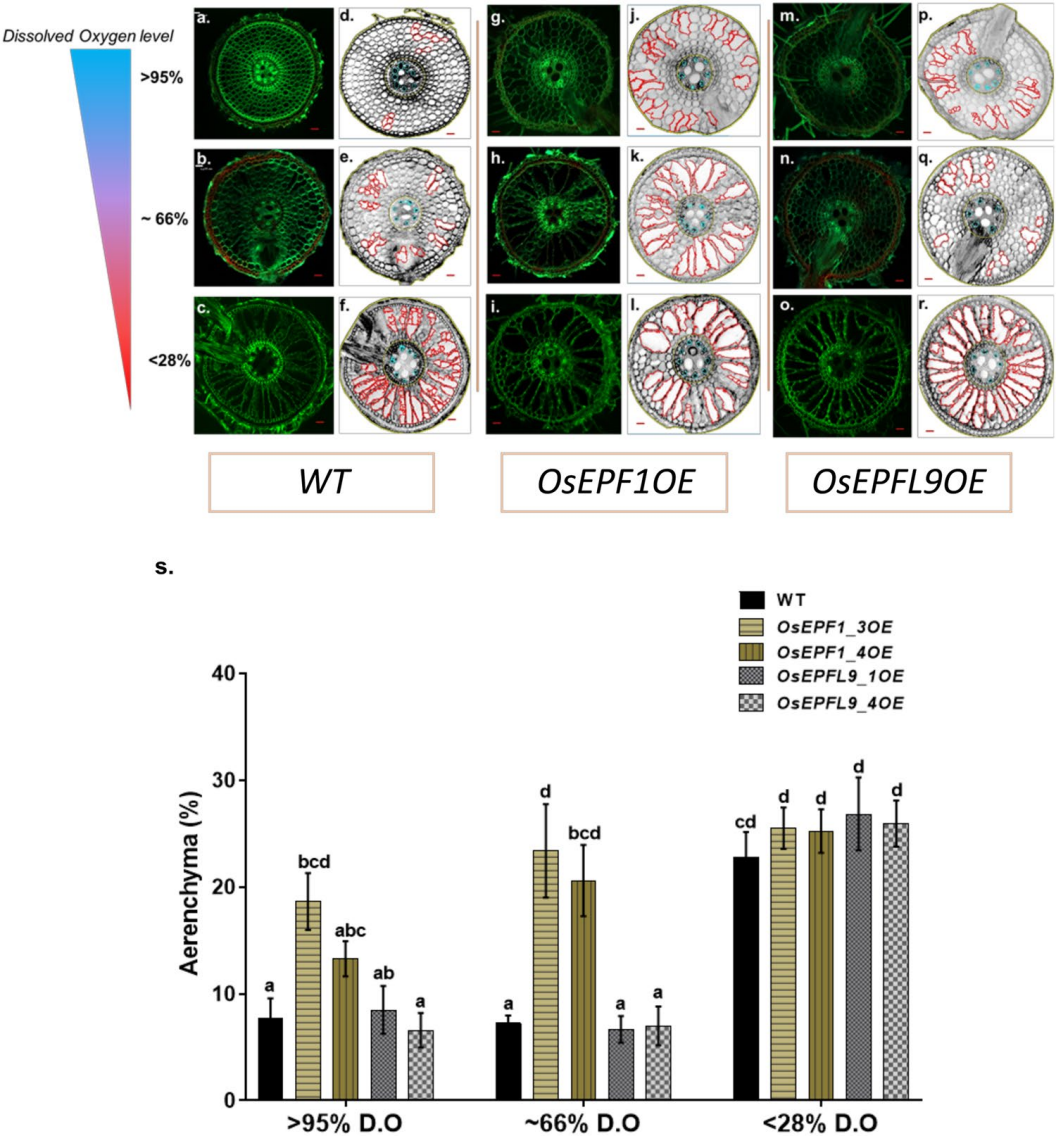
Dissolved Oxygen levels impact aerenchyma formation. The adventitious root section of the 35-day old plant roots were sampled at 6 cm from the root tip. a-r. The RCA formation (**a**–**c**,**g**–**i**,**m**–**o**) and estimation of aerenchyma via Rootscan 2 (**d**–**f**,**j**–**l**,**p**–**r**). WT (**a**–**f**), *OsEPF1*OE (**g**–**l**) and *OsEPFL9*OE (**m**–**r**) roots at three different dissolved oxygen levels (95%, 66%, and 28%). (**s**) Bar graph showing estimated RCA at varying D.O levels for WT, two Os*EPF1*OE lines and two* OsEPFL9*OE lines. Different letters indicate significant differences between the lines (p < 0.05). All values are represented as the mean ± SEM. N = 8. Scale bar = 50 µm.

To fully understand how aeration can influence RCA formation, WT, *OsEPF1*OE and *OsEPFL9*OE lines were grown hydroponically in aerated, semi-aerated and non-aerated liquid media (Fig. 7). In the aerated tubs the mean dissolved oxygen (D.O.) level remained at 95 (±2)% throughout tissue sampling, while in the semi aerated conditions oxygen level dropped to 66 (±5)% and the non-aerated condition to 28 (±2)% by week 5. The temperature of the aerated hydroponic tubs (28.7 °C ± 0.1) was significantly lower (P = 0.0109) than the mean temperature of the oxygen-deficient condition (29.4 °C ± 0.2) (Supplementary Fig. 6).

**Figure 8:**
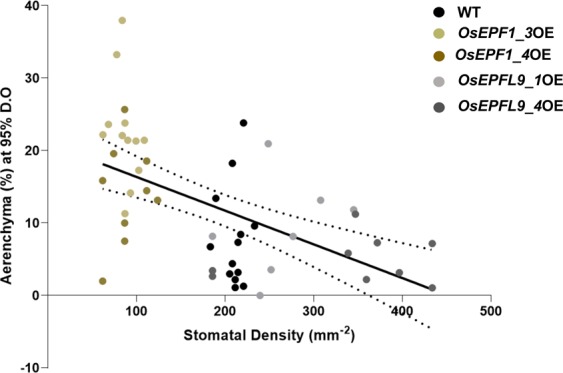
Stomatal density is quantitatively related to root aerenchyma formation. The linear regression of 5-week old plants between the estimated aerenchyma at 95% D.O level and the abaxial stomatal density of the WT, *OsEPF1*OE, and *OsEPFL9*OE in aerated condition. (P = 0.0001 and R^2^ = 0.3020). N = 7–12.

Analysis of air space under aerated (95% D.O) and semi-aerated (66% D.O) conditions revealed that compared to WT controls, *OsEPF1*OE roots had 56.7% and 68.9% more air space under the same conditions (P = 0.0169 and P < 0.0001) (Fig. 7a–e,g–k,s). On the other hand, *OsEPFL9*OE plants had similar air space values as WT under both treatments (Fig. 7a–e,m–q,s). Under oxygen-deficient conditions (28% D.O), WT and *OsEPFL9*OE plants increased air space to compensate for low oxygen content in the root medium, but *OsEPF1*OE plants did not (Fig. 7c,f,i,l,o,r,s). These results indicate that while both WT and *OsEPFL9*OE roots adjust their RCA air spaces in responses to water oxygen content, *OsEPF1*OE do not. Under aerated conditions when there are differences in aerenchyma formation a statistically significant (P = 0.0001 and R^2^ = 0.3020) negative correlation between stomatal density and % air spaces was detected (Fig. 8).

## Discussion

Here we show that overexpression of *OsEPF1* in rice results in significantly reduced stomatal density, *E* and *g*_*s*_, consistent with similar results from rice^4^ and other species such as Arabidopsis and barley^4,31,51^. We reveal evidence for enhanced water use efficiency and greater soil water conservation resulting primarily from the aforementioned lower rates of *E* and *g*_*s*_4. Importantly, the reduced stomatal density of *OsEPF1* overexpression has a proportionally greater effect on *g*_*s*_ than on *A*, or any substantial reduction in carbon gain.

Here we unexpectedly observed that *OsEPF1* overexpression lines have increased root cortical aerenchyma. Aerenchyma are thought to form in roots during low-oxygen soil conditions to aid oxygen diffusion across plant tissues, in addition to other mechanisms that increase oxygen levels in roots, such as the reduction in Radial Oxygen Loss (ROL) to the soil environment^56^. Despite the importance of aerenchyma tissue to root function in waterlogged soils and high irrigation agriculture, little is known about how its development is regulated^57^. Unlike other monocots, lysigenous aerenchyma in rice can form constitutively in mature root tissue under aerobic conditions with the formation toward the root tip being inductively enhanced in response to oxygen deficiency (e.g. waterlogged soil)^26,28,58^.

Aerenchyma formation has also been observed in roots subjected to drought^24,59,60^. In maize, loss of aerenchyma has also been correlated with improved growth and grain yield under low P conditions^61^. The formation of aerenchyma in response to such varied abiotic stimuli (and indeed in different tissues) suggests that it may fulfil more than one function. For example, the loss of cortical tissue might also act to reduce respiratory loss of carbon reserves at a time when photosynthate supply is limited^24,59,60^. The multifunctional nature of aerenchyma raises new questions concerning the importance of each stimulus, their interaction and the signalling networks involved.

What is the mechanism for the constitutive formation of aerenchyma tissue in *OsEPF1*OE lines? One explanation may lie in the possible direct role of *OsEPF1* in the regulation of signalling processes in the roots. The transgene overexpression in this study was directed by a constitutive promoter, so it is possible that ectopic *OsEPF1* expression induced a response directly involved with the development module associated with aerenchyma formation. Indeed, *OsEPF1* was highly expressed in the roots of *OsEPF1*OE lines confirming the potential for *EPF1* to alter aerenchyma in our study (Fig. 1 and Supplementary Fig. 8). In Arabidopsis roots, the EPFL9-ERECTA module has been shown to regulate cortex cell proliferation by regulating the activity of SPINDLY^34^. In rice *OsEPF1* has also been shown to be upregulated in the roots of a sodium-sensitive rice cultivar (IR29), but not in a sodium tolerant rice cultivar (FL478), on exposure to salt-stress (supplementary data in Senadheera *et al*.^62^). These suggest the potential for *EPFs* to impact on root stress signalling at the local level.

To test whether potential EPF signalling module components are expressed in rice root tissues, we tested the levels of expression of genes known to be involved in stomatal development and patterning including *OsER* (Os06g10230), *OsER2* (Os02g53720), *OsERL1* (Os06g03970) and *OsTMM* (Os01g43440) in both WT and *OsEPF1OE* plants. We found almost undetectable levels of these transcripts in the roots of all the plants surveyed (Supplementary Fig. 8). Based on this lack of expression of these key genes, we conclude that it is highly improbable that the observed changes in aerenchyma formation in the *OsEPF1 *over-expression lines (or through translocation of EPF peptides from shoot to the root) occur via EPF1- ER/ER2/ERL1-TMM signalling.

What other signalling pathways could interact to produce the constitutive aerenchyma phenotype? Reactive oxygen species are formed in the root in oxygen-deficient conditions and they have also been implicated in aerenchyma formation^63^. In Arabidopsis, ROS have been implicated in regulating cortex cell proliferation by regulating the activity of SPINDLY through an EPFL-ERECTA module^34^. However, we exclude this possibility as *i*) we did not find detectable expression of *ERECTA* and *ERECTA*-like genes in rice roots as discussed above and *ii*) aerenchyma formation is a distinct and different process to cell proliferation. In wheat, ROS production is ethylene-mediated; ethylene induces expression of genes involved in ROS synthesis and pre-treatment with diphenyleneiodonium (DPI- inhibitor of NADPH oxidase- a key enzyme in ROS synthesis) inhibits ethylene mediated aerenchyma formation. However, we did not find any differences in the expression of NADPH oxidase between WT and OE lines, nor between different oxygenation treatments so we suggest that ROS are unlikely to regulate aerenchyma formation in the *EPFOE* lines (Supplementary Fig. 9).

In sugarcane, the ethylene-auxin balance has been implicated in root aerenchyma formation^64^. Thus, one possible mechanism could be through regulation of auxin transport and response in the roots to alter auxin ethylene balance. Auxin transport and activity have been implicated in stomata development^65^. Auxin response factor MONOPTEROS (*ARF5*) has been shown to bind directly to the *STOMAGEN*/*EPFL9* promoter and represses its expression to negatively regulate stomata development^66^. However, such a response in our overexpression lines is unlikely as the *EPF/L* genes are driven by the exogenous maize ubiquitin promoter. Nonetheless, overexpression of *EPF2* in Arabidopsis has been shown to diminish the auxin response during leaf margin morphogenesis further linking *EPF/L* and auxin function^67^. At this stage, we cannot rule out a mechanism involving the interplay of auxin, ethylene and EPF/L signalling in regulating root aerenchyma formation.

There may be another mechanism based on physiological constraints to gas diffusion. For example in Arabidopsis, plants overexpressing the *OsEPF1* orthologue, *EPF2* have been shown to have smaller root areas, which the authors attribute to reduced *g*_*s*_^8^, suggesting that changes in root development could be driven by changes in plant physiology associated with changes in reduced gaseous diffusion via stomata. The predominant route for oxygen transport (in low-oxygen soils) is from the atmosphere into the leaf and shoot tissue via stomatal pores, from where it diffuses towards the roots, aided by the airspaces formed in the root cortex. To assist this there are also air spaces within the leaf midrib, true stem (culm) and the leaf sheath^68^. It may be possible that in our *OsEPF1OE* lines, a reduction in *g*_*s*_ as a result of the lowered stomatal density has formed a resistance to oxygen diffusion within the plant, stimulating aerenchyma formation potentially via the ethylene signalling pathway^28,58,69–72^. Such a mechanism would however be dependent on the roots being unable to absorb large quantities of O_2_ directly from the hydroponic media. If this was the case, *OsEPFL9*OE plants may have shown the opposite phenotype (Fig. 7) but in fact the *OsEPFL9*OE RCA phenotype observed was closer to *OsEPF1*OE than WT plants under aerated conditions. Ethylene is implicated in the formation of lysigenous aerenchyma in rice, wheat and maize^28,58,72^ where it accumulates in low oxygen conditions strongly inducing more aerenchyma in both the apical and the basal regions^58^. Ethylene treatment to roots has been shown to result in further increase in the formation of aerenchyma in rice under aerated conditions^28^ whereas treatment with ethylene perception inhibitors (e.g. silver ions) decreased the formation of aerenchyma. The treatment of wheat and maize roots with ethylene biosynthesis inhibitors amino ethoxy vinyl glycine, amino-oxyacetic acid, and cobalt chloride blocks the formation of aerenchyma in low-oxygen condition^69–71^. However, there is currently no direct evidence linking EPF signalling and ethylene-mediated aerenchyma formation.

Finally, and as noted above aerenchyma has been implicated in productivity by reducing respiratory ‘load’ of root tissue. However, it is unclear whether this has bearing on the interpretation of our data. The root biomass data suggests that, if the physiological hypothesis is correct, then the supposed increased access to oxygen was not conferring a growth advantage to the transgenic lines in these experiments.

## Conclusions

Plant biology has often studied physiological and molecular events of above ground and below ground development separately in mature plants, even though many examples of whole plant signalling are known^73^. This is in part due to the difficulty of studying root processes with the same ease as shoots. Recent advances in methodology should enable better understanding of many signalling processes and physiological mechanisms coordinating root and shoot function^74^. Here we report a novel observation, that the expression of a gene with a key role in the regulation of leaf stomatal development and pattering (Os*EPF1*) also influences the development of root aerenchyma tissue when over-expressed.

*EPF1* is a well characterised gene involved in regulation of stomatal patterning. Rice plants that had elevated levels of *OsEPF1* showed effects on stomatal density, stomatal size, stomatal conductance, leaf water use efficiency and drought tolerance. However, we also showed O_2_-independent aerenchyma formation in the root tips which is normally induced by growth in oxygen-deficient or waterlogged conditions. Although the mechanism is currently unknown, these data have important implications for mechanisms of the transport of oxygen from shoot to root and in the signalling of oxygen deficiency in root tissue.

## Supplementary information

Supplementary figures and table
