# Author Correction: Rice plants overexpressing *OsEPF1* show reduced stomatal density and increased root cortical aerenchyma formation

**DOI:** 10.1038/s41598-019-51402-7

**Published:** 2019-10-10

**Authors:** U. Mohammed, R. S. Caine, J. A. Atkinson, E. L. Harrison, D. Wells, C. C. Chater, J. E. Gray, R. Swarup, E. H. Murchie

**Affiliations:** 1 Division of Plant and Crop Science, School of Biosciences, University of Nottingham, Sutton Bonington campus, LE12 5RD Nottingham, UK; 2 Department of Molecular Biology and Biotechnology, University of Sheffield, Western Bank, S10 2TN Sheffield, UK

Correction to: *Scientific Reports* 10.1038/s41598-019-41922-7, published online 03 April 2019

The Acknowledgements section in this Article is incomplete.

“This work was supported by the Biotechnology and Biological Sciences Research Council [grant numbers BB/R004633/1, BB/N021061/1, BB/N013646/1]. JAA and DMW receive funding from the University of Nottingham Future Food Beacon of Excellence. We thank Dr Amanda Rasmussen (University of Nottingham) for assistance with the oxygen sensor.”

should read:

“This work was supported by the Biotechnology and Biological Sciences Research Council [grant numbers BB/R004633/1, BB/N021061/1, BB/N013646/1 and BB/P010520/1]. JAA and DMW receive funding from the University of Nottingham Future Food Beacon of Excellence. We thank Dr Amanda Rasmussen (University of Nottingham) for assistance with the oxygen sensor.”

