# Supplementary material for: Rice plants overexpressing OsEPF1 show reduced stomatal density and increased root cortical aerenchyma formation
Source: Sci Rep. 2019 Apr 3;9:5584. doi: 10.1038/s41598-019-41922-7 (PMC6447545; doi:10.1038/s41598-019-41922-7)
Supplement: Supplementary file 1 — Supplementary figures and table [file 41598_2019_41922_MOESM1_ESM.pdf]

Rice plants overexpressing *OsEPF1* show reduced stomatal density and increased root cortical aerenchyma formation

Mohammed U[1], Caine RS[2], Atkinson JA[1], Harrison EL[2], Wells D[1], Chater CC[2], Gray JE[2], Swarup R[1], Murchie EH[1]*

[1] Division of Plant and Crop Science, School of Biosciences, University of Nottingham, Sutton Bonington campus, LE12 5RD

[2] Department of Molecular Biology and Biotechnology, University of Sheffield, Western Bank, S10 2TN

*Correspondence to: erik.murchie@nottingham.ac.uk

Gene	Primer sequence	
	Forward	Reverse
OsEPF1 (Os04g54490)	TTGTGATCTCACCACAGGCCACAG	TTGAAGCTCACCATGACGCGGTTG
OsEPFL9 (Os01g68598)	CATGGCAGCATCTCAGGTACAG	TGCATATTTGGGCAGCCTCTCC
OsER (Os06g10230)	GTTGAAGGGCATCGTCTCGATTG	GCCAATCTCATCAGGGATCTGC
OsER2 (Os02g53720)	GGCTGGCATTGTACCAACAGAC	CCAGGATTACCCAAGAAGCTGTCC
OsERL1 (Os06g03970)	CAACCACAGCCACTTGTCAAAGG	ACAACTAGCTTTGGAGGTCCTTGC
OsTMM (Os01g43440)	AACAACCTCACATCCGCCATCC	TGTAGCTCAGGTCGAGAAGCTG
OsPROFILIN (Os06g05880)	GGTTGTCATCCGAGGAAAGAAGGG	ACGACAGGCCAGTCTTCTTGAC
OsTFIIEβ (Os10g25770)	GTGCAGCCCAAGGCTAAG	CGTCGAATAAGCGTAGAGCA
OsRBOHH (Os12g35610)	GTTCGACGTCATGGGCTACT	TTGATGTAGAGGCAGACGCC
OsCDPK5 (Os02g46090)	GCTACGGACCAGAAGCTG	GCAATCTCCTCCTCTGAAAGGC
**Actin**	CGGGAAATTGTGAGGGACAT	AGGAAGGCTGGAAGAGGACC

Supplementary table 1. List of primers used for gene expression studies

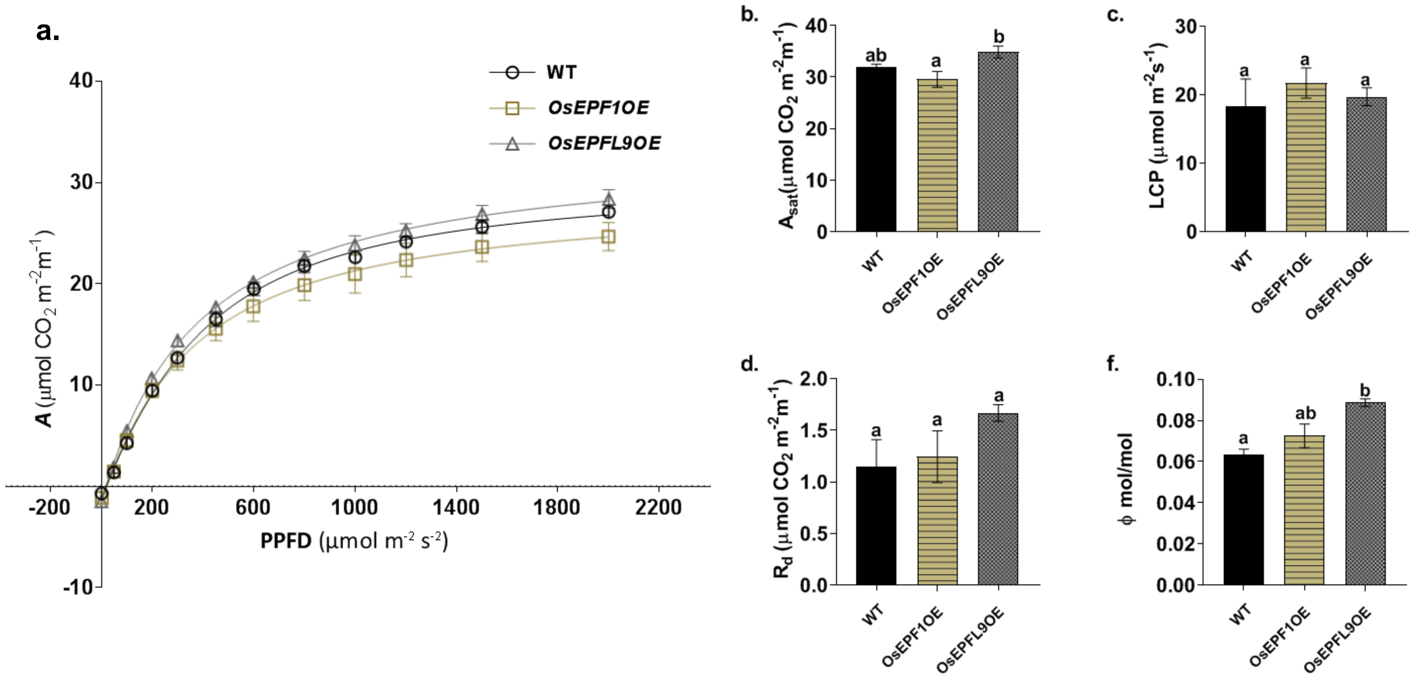

Supplementary figure 1. Altering stomatal density does not change photosynthesis.
The effect of stomatal density on net photosynthetic rate (*A*) response to photosynthetic photon flux density (PPFD) and estimated parameters derived from the fitted light response curve (LRC) as described by a four-parameter non-rectangular hyperbola. **a**. Photosynthetic CO_2 assimilation (*A*) **b**. The net assimilation rate at saturated light (A_{max}). **c**. Light compensation point (LCP). **d**. (Mitochondrial) dark respiration rate (R_d). **d**. Apparent quantum yield. All values are means ±SE. Different letters represent significant differences among the genotypes. N = 3-4. These experiments were conducted on *OsEPF1*OE line 3 and *OsEPF9*OE line 1 showing the strongest phenotypes.

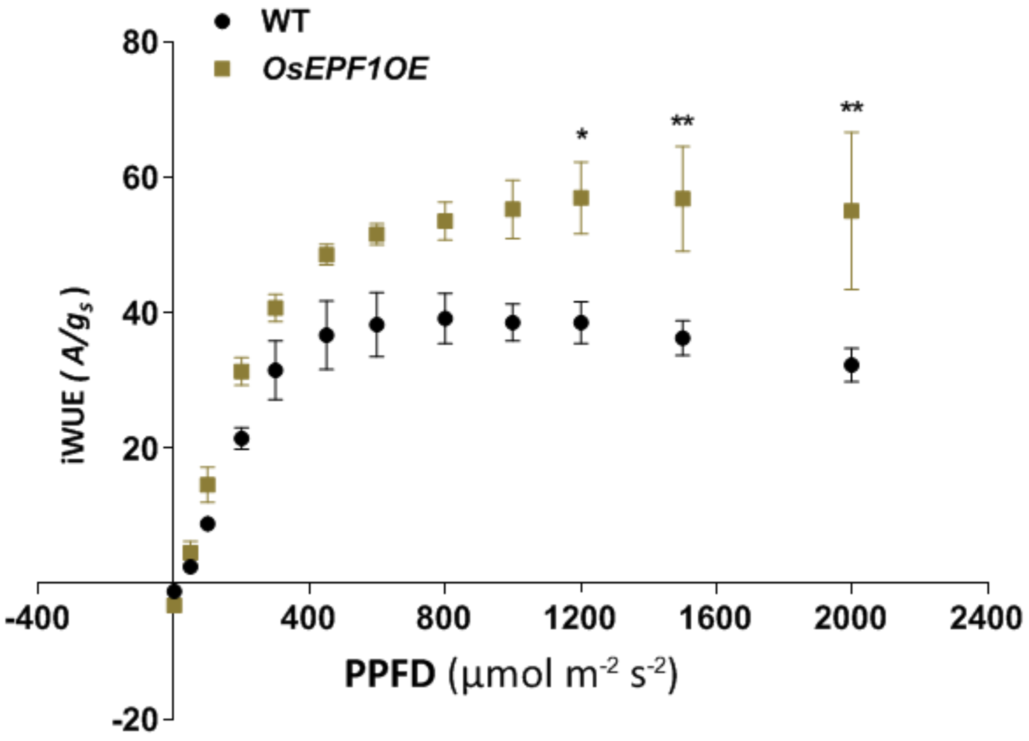

Supplementary figure 2. **Reducing stomatal density improves leaf water use efficiency.**
The response of intrinsic water use efficiency (iWUE, *A/gs*) to photosynthetic photon flux density (PPFD). Each data point represent the mean ± Standard Error of the Mean (SEM). N = 3. Asterisk represent statistical differences at each light point (*$P < 0.05$, **$P < 0.01$). These experiments were conducted on *OsEPF1*OE line 3 showing the strongest phenotypes.

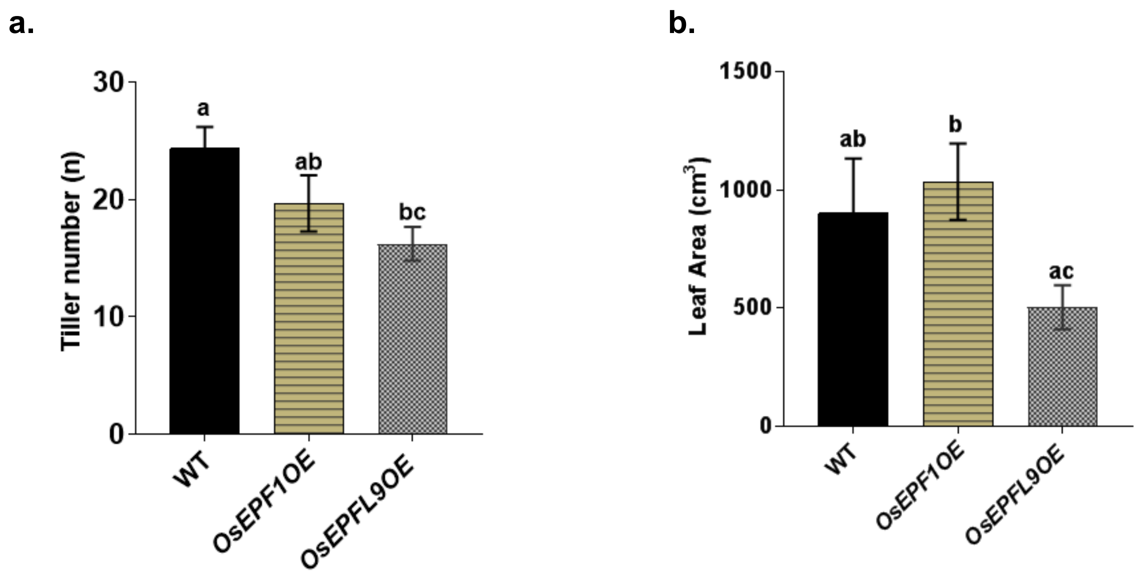

Supplementary figure 3. Reduction of stomatal density does not affect the changes in shoot biomass properties.

a. Number of tillers in 8 week old plants **b.** Total leaf area at booting stage. Leaf area was measured with a Li-3100C (Licor Inc, Licoln, Nebraska). Letters represent significant differences between the genotypes (P<0.01). For this experiment the strongest phenotype over expression lines were used for each genotype. These experiments were conducted on *OsEPF1*OE line 3 and *OsEPF9*OE line 1 showing the strongest phenotypes. Error bars indicate the Standard Error of the Mean (SEM). N = 6-11 plants (single leaf per plant).

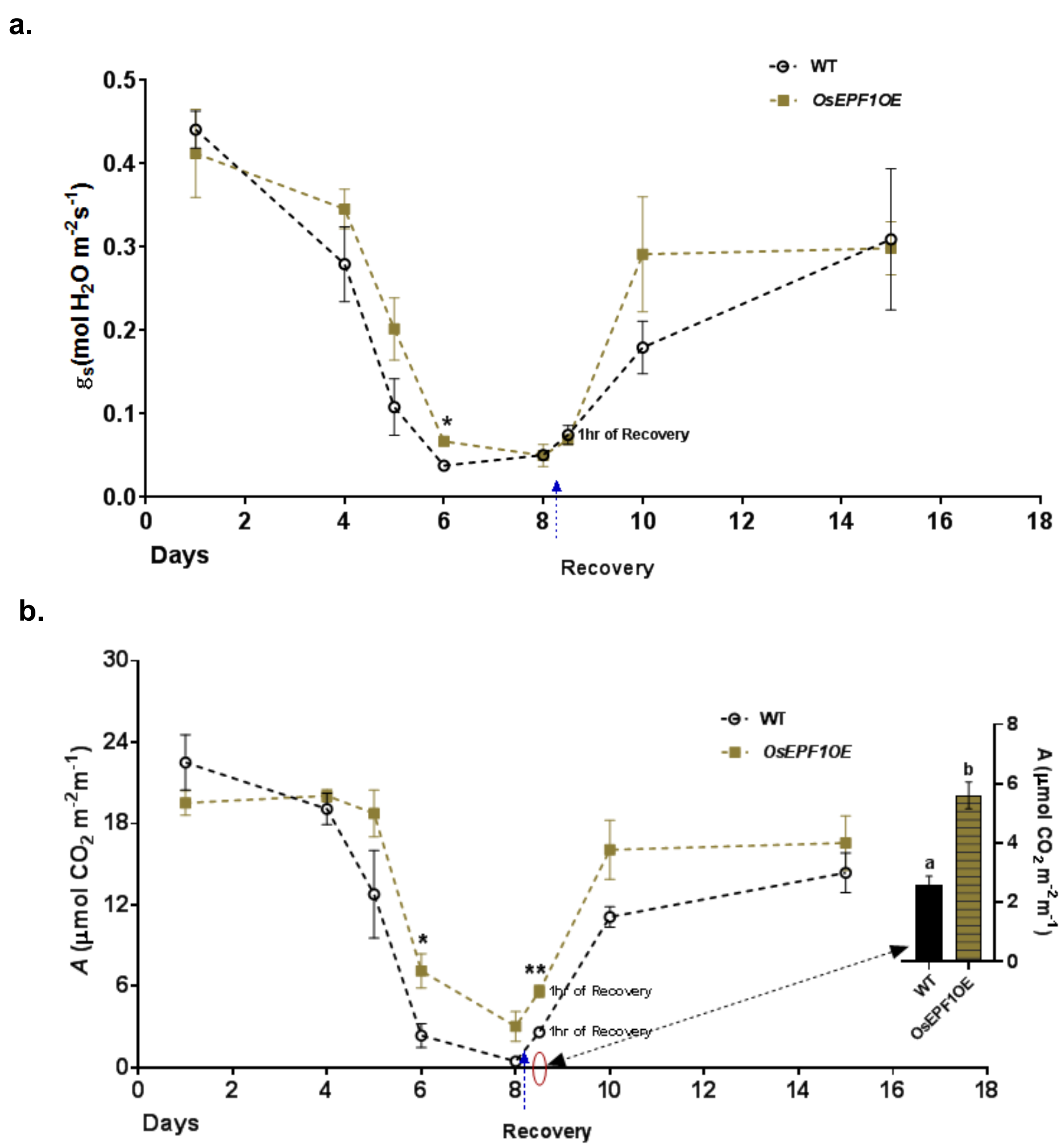

Supplementary figure 4. Reduction of stomatal density enables plants to maintain photosynthesis for longer duration.

Time-course of the effect of drought stress between the WT and the *OsEPF1*OE in 5-week old plants . **a**. stomatal conductance (*gs*). **b**. CO_2 assimilation rate (*A*). The asterisks represent the significant differences between the two treatments at the same time-point. (Student T- test; $*P \leq 0.05$, $**P \leq 0.01$). Error bars indicate the Standard Error of the Mean (SEM). N = 3-7. These experiments were conducted on *OsEPF1*OE line 3 showing the strongest phenotypes.

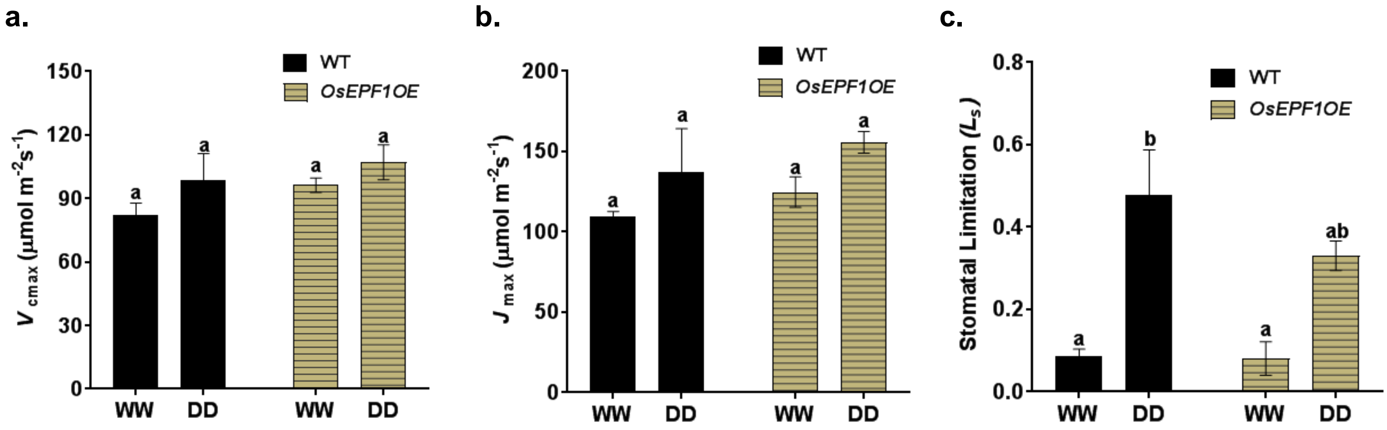

Supplementary figure 5. **Reduction in stomatal density does not alter the photosynthetic biochemistry.**

Summary of the *A*/*C*i curve estimates of the photosynthetic biochemical and non-biochemical components of WT and *OsEPF1*OE between drought and non-drought. **a.** The maximum carboxylation rate of Rubisco (V_{cmax}). **b**. The maximum rate of RuBP regeneration (J_{max}). **c**. The stomatal limitation imposed on *A* (L_s). Different letters indicates significant differences between treatments and across genotypes ($P < 0.05$). All values are means with SEM error bars. N = 3-4.

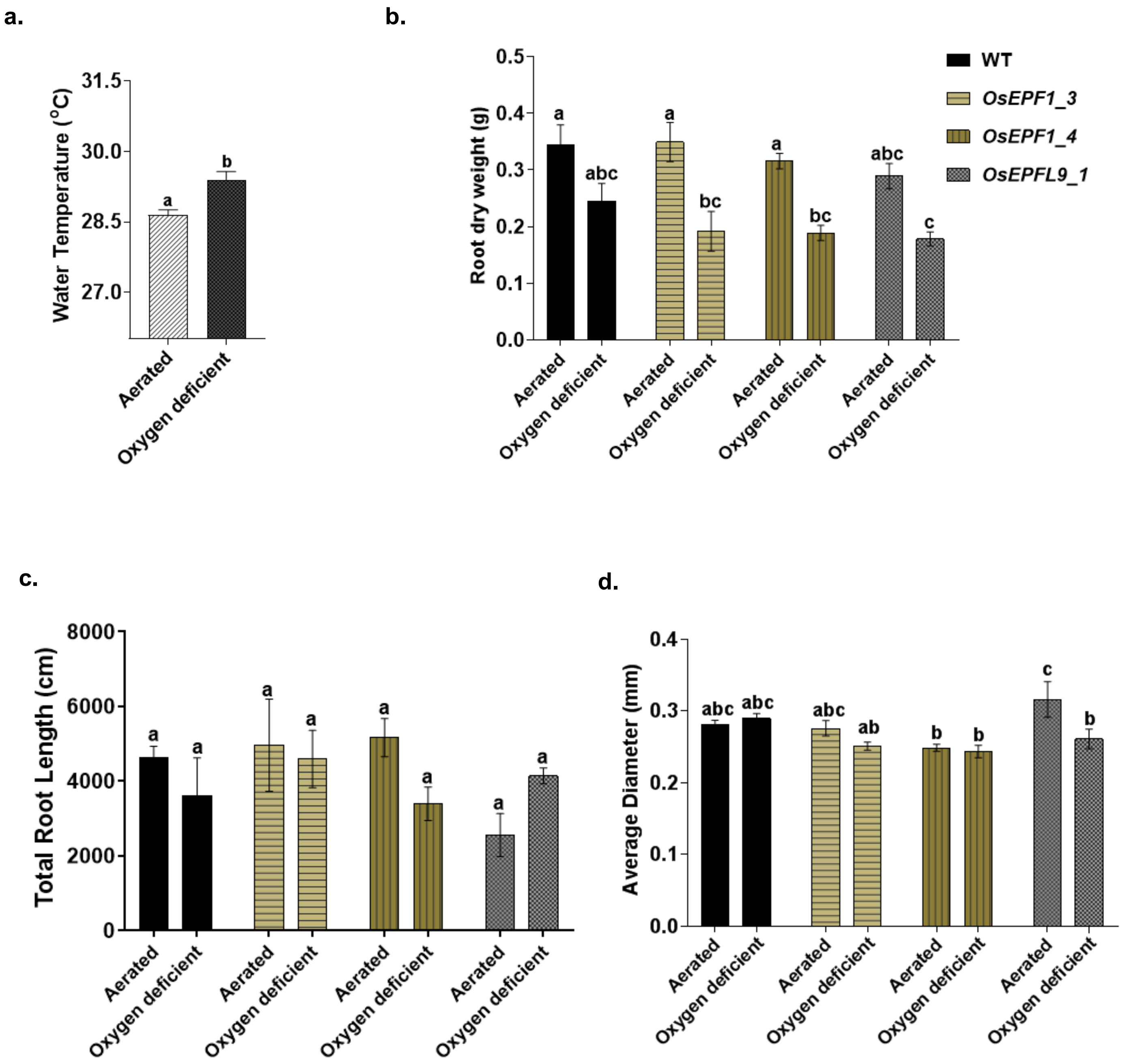

Supplementary figure 6. Root phenotypic analysis of O*sEPF* lines under aerated and oxygen deficient conditions show no significant differences from WT plants.

a. The temperature of the hydroponic media. **b.** The root total dry weight. **c.** Total root length (total length of all roots, including the lateral roots as computed by winRhizo). **d.** Average root diameter. Different letters indicate significant differences between lines ($P < 0.05$). All values are represented as the mean ± SEM. N = 5-7 .

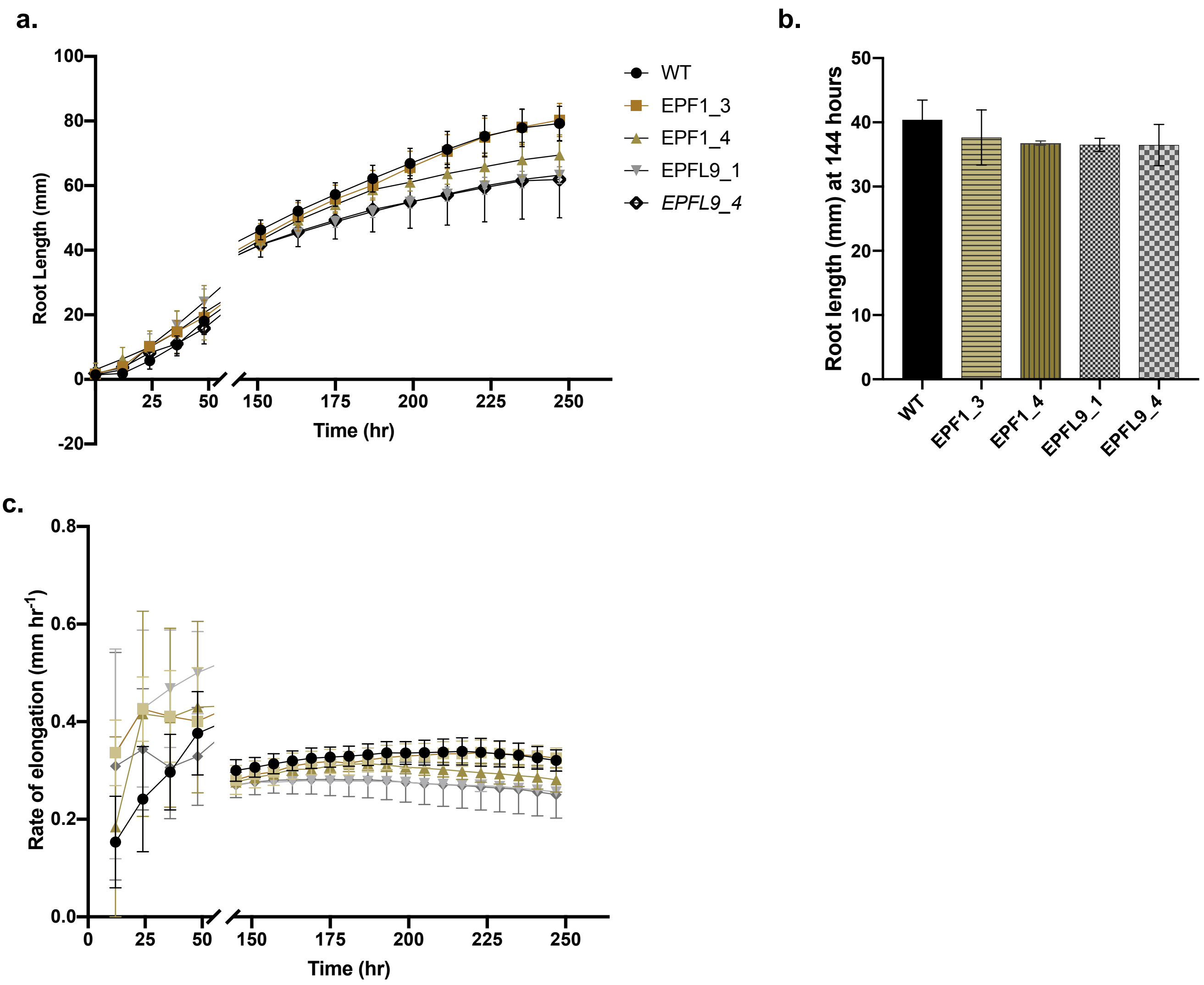

Supplementary figure 7. Root phenotypic analysis show that root growth rates and root elongation rates of *OsEPF1*OE and *OsEPFL9*OE lines are comparable to WT plants.
a. Measurement of seminal root length over time. **b.** The root length at 144 hours. **c**. The hourly rate of root elongation. The roots were 5 day old when the measurements commenced. Each data point represented as the mean and error bars indicate ± SEM. There were no significant differences between any lines at any point ($P<0.05$). Growth conditions: Temp 23°C; 12 hours day/night.

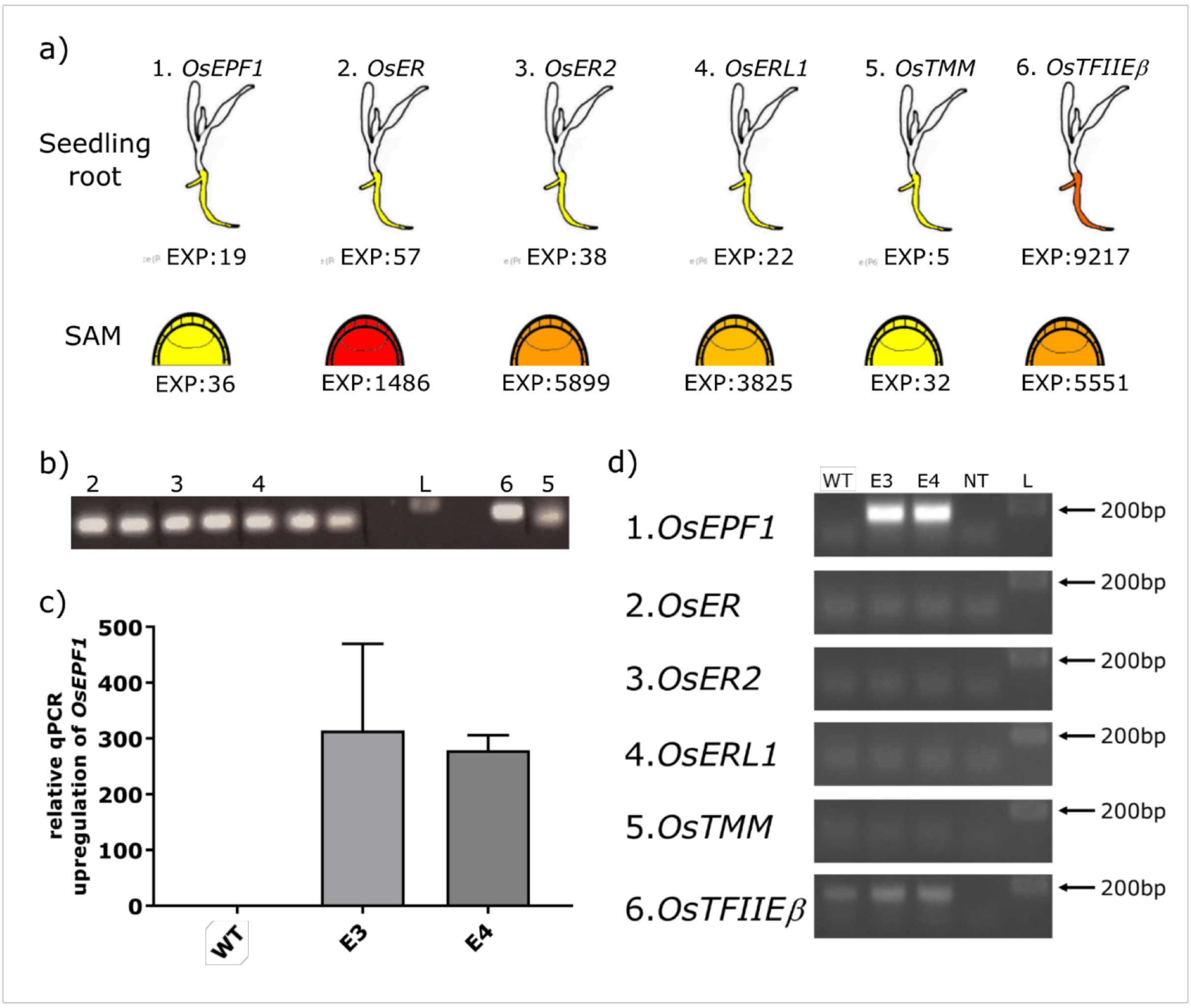

Supplementary figure 8. Genes encoding targets for EPF1 peptide show no or very low expression in the roots of WT and *OsEPF1*OE lines suggesting over-expression of *OsEPF1* does not induce expression of other stomatal patterning genes in the roots.

a. Seedling root and shoot apical meristem (SAM) Electronic Fluorescent Pictograph (eFP) images of rice *OsEPF1, OsER*, *OsER2*, *OsERL1*, *OsTMM* and the qRT-PCR control gene *TRANSCRIPTION INITIATION FACTOR IIE SUBUNIT BETA* (*OsTFIIβ*) (Jain et al., 2007; Winter et al., 2007; Shiono et al., 2014). All eFP image profiles were set to an absolute value of 15,000, with actual expression values listed underneath. Red indicates relatively high gene expression, orange indicates a medium level of expression and yellow, low to very low expression. **b.** Gel electrophoresis image of RT-PCR reactions (40 cycles) showing the above ground expression levels of *OsER* (2), *OsER2* (3), *OsERL1* (4), *OsTMM* (5) and *OsTFIIEβ* (6) in 8 day old Nipponbare seedlings. Position of 200bp marker can be seen in the DNA ladder (L). Note. For each primer pair, q-RTPCR validation testing showed that levels of gene expression were consistent across a dilution series of seedling RNA. ***c.*** Relative expression level of *OsEPF1* in the 37 day old roots of WT and *OsEPF1*OE (Line 3 and Line 4*)* plants grown in aerated hydroponic media showing high expression compared to control plants (WT). N = 2-3. **d.** RT-PCR analysis confirms that *OsERECTA (OsER), OsER2, OsER-like 1 (OsERL1)* and *OsTMM* are not expressed in the roots of the Wild type control plants (WT) and *OsEPF1* over-expressing plants E3 and E4. NT denotes no template control and L denotes DNA ladder. *OsTFIIEβ* was used as a reference gene showing similar expression in WT and *EPF1OE* lines 3 and 4.

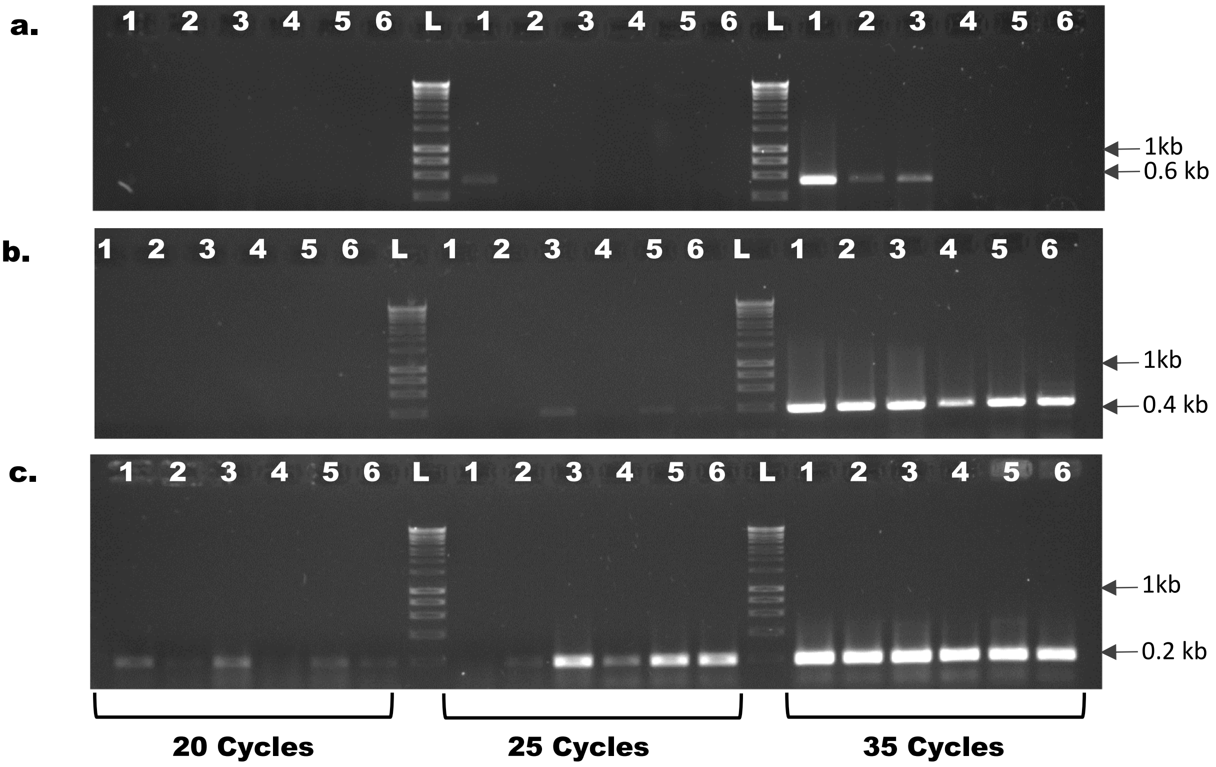

Supplementary figure 9. Reactive oxygen species (ROS) are unlikely to regulate aerenchyma formation in *EPF1*OE lines.

Expression of **a**. *RESPIRATORY BURST OXIDASE HOMOLOG H (OsRBOHH)* and **b.** *CALCIUM-DEPENDENT PROTEIN KINASE* (*OsCDPK5)* was analysed in the roots of 37 day old rice plants grown in aerated hydroponic media by semi-quantitative RT-PCR over 20, 25 and 35 cycles. Rice *Actin* gene was used as a control (**c**) . Lane 1= WT root, lane 2= *OsEPF1_3*OE root, lane 3= *OsEPF1_4*OE root, lane 4= WT leaf, lane 5= *OsEFP1_3*OE leaf and lane 6= *OsEPF1_4*OE leaf. L= 1 kb Hyper ladder. Expected band sizes: *OsRBOHH ~538bp; OsCDPK5 ~400bp; Actin ~200* bp.
